# Advantages of a Multi-Platform Workflow Integrating Biphasic Extraction, Chromatography, and NMR for Characterization of Metabolites in Extracts of *Malakabi* Ajwa Date Seeds

**DOI:** 10.3390/metabo16080552

**Published:** 2026-08-04

**Authors:** Mufeed M. Basti, Shanelle Flowers, Anika R. Barnes, Leonard L. Williams, Daniel A. Todd, Kiran Subedi, Amira A. Ayad

**Affiliations:** 1 Department of Chemistry, North Carolina A&T State University, Greensboro, NC 27411, USA; 2 Reynolds Tobacco Company, Winston Salem, NC 27105, USA; shanelle_flowers_external@rjrt.com; 3 The University of North Carolina at Chapel Hill, Chapel Hill, NC 27514, USA; arba@unc.edu; 4 Center for Excellence in Post-Harvest Technologies, NC Agricultural and Technical State University, Kannapolis, NC 28081, USA; llw@ncat.edu; 5 Procter & Gamble, Mason, OH 45040, USA; todd.da@pg.com; 6 Analytical Services Laboratory, North Carolina A&T State University, Greensboro, NC 27411, USA; ksubedi@ncat.edu

**Keywords:** *Malakabi* Ajwa date seeds, biphasic extraction, metabolite profiling, GC-MS, LC-MS, NMR spectroscopy, orthophosphate monoesters

## Abstract

**Background/Objectives**: Date seeds contain a wide range of biologically active constituents and have been associated with multiple health benefits. Our study aimed to evaluate the utility of biphasic aqueous methanol/chloroform extraction for separating diverse metabolites from powdered *Malakabi* Ajwa date seeds and to identify the most effective chromatographic and spectroscopic techniques for their comprehensive profiling and structural characterization. **Methods:** Biphasic extraction generated organic, aqueous, and insoluble phases. Proper chromatography-mass spectrometry and spectroscopy analytical tools were then utilized to profile the metabolites in the aqueous and organic phases. **Results and Conclusions:** In the organic phase, fatty acids and their methyl esters were identified using GC-MS and confirmed by ^1^H NMR, while the absence of an O–H band in the FT-IR spectrum, together with ^1^H NMR data, supported the natural esterification of these fatty acids. In the aqueous phase, UV-Vis spectroscopy and LC-MS revealed anthocyanin pigments, and LC-MS additionally detected four amino acids. HPLC was used to quantify and validate the sugar composition, and ^1^H 1D and 2D NMR spectra enabled the assignment of α- and β-glucopyranose, β-fructopyranose, and sucrose signals. ^31^P NMR indicated that orthophosphate monoesters were the predominant phosphorus-containing compounds in both organic and aqueous phases. Overall, our findings show how an integrated chromatographic and spectroscopic workflow provides a robust strategy for comprehensive metabolite profiling of natural products such as *Malakabi* Ajwa date seeds.

## 1. Introduction

Date palm (*Phoenix dactylifera* L.) is a globally important crop valued for its nutritional and economic significance. In 2018–2019, production in Riverside County, California, alone was ~29 million pounds. Beyond the carbohydrate, fiber, vitamin, and mineral content of the fruit, date seeds have also attracted attention for their nutritional and economic potential [1].

*Malakabi* Ajwa date seeds are a valuable by-product rich in essential minerals such as potassium, magnesium, calcium, and phosphorus [2], along with dietary fiber, proteins, phenolic compounds, and bioactive oils [3,4]. Owing to this diverse composition, they have promising applications in fertilizers, animal feed, food products, and cosmetics. The presence of phenolics and bioactive oils suggests potential human health benefits, particularly through antioxidant and anti-inflammatory activities [5].

Metabolite profiling is a key tool for studying plant physiology and responses to environmental change. It has been applied in areas such as herbicide diagnostics in barley, monitoring tomato ripening, and comparison of genetically modified versus conventional crops [6]. A range of analytical platforms, including HPLC, NMR, LC-MS and GC-MS, are widely used in such studies. Each method, however, has inherent limitations. GC-based techniques require volatile or derivatized analytes, LC-MS relies on efficient ionization, and NMR typically needs relatively high analyte concentrations and can suffer from signal overlap [7,8,9]. These constraints motivate the use of complementary analytical approaches. Among these platforms, NMR remains one of the most informative tools for metabolomics. It enables simultaneous detection of diverse metabolites and inherently quantitative comparisons, because signal intensity is proportional to molar concentration. NMR-based metabolomics has been successfully applied to various functional foods, including legume sprouts. Since carbohydrates, amino acids, and fatty acids are known constituents of date seeds, combining chromatographic and spectroscopic techniques is particularly suitable for detecting and characterizing these metabolites [10,11].

Mass spectrometry and NMR, when coupled with chromatographic separation, provide a powerful platform for comprehensive metabolite profiling. MS delivers highly sensitive detection and elemental composition, whereas NMR provides detailed information on structural moieties; together, they greatly enhance confidence in compound identification. For example, glucopyranose can be detected and assigned a molecular formula by MS, while ^1^H NMR spectra resolve its α and β anomeric forms (Figure S1), offering structural detail that MS alone cannot provide [12].

*Malakabi* Ajwa date seeds have not yet been comprehensively profiled using modern metabolomic approaches, and previous studies on date seeds have generally relied on only one or two analytical platforms. In this work, we combine biphasic aqueous methanol/chloroform extraction with multiple chromatographic and spectroscopic techniques namely, LC-MS, GC-MS, HPLC, FT-IR, and 1D/2D ^1^H and ^31^P NMR, within a single integrated workflow. This strategy enables simultaneous detection of sugars, fatty acid esters, amino acids, pigments, and phosphorus-containing metabolites across both aqueous and organic phases. Also in many cases, it allows for structural confirmation of key metabolites such as sugar spin-system assignments by two-dimensional ^1^H NMR, verification of naturally esterified fatty acids through complementary FT-IR, GC-MS and NMR data, and characterization of phosphorus-containing metabolites using ^31^P NMR, which has not been previously reported for Ajwa date seed extracts. Overall, our study aims to assess the benefits of this biphasic, multi-platform approach for metabolite profiling in *Malakabi* Ajwa date seeds, to evaluate which metabolite classes are most effectively detected with each method, and to demonstrate how integrating these complementary tools enhances metabolome coverage in natural product metabolomics.

## 2. Materials and Methods

### 2.1. Preparation of Solvent Extracts

*Malakabi* Ajwa dried date seeds were obtained from a certified date supplier in Aswan City, Egypt. Following the procedures described by Fiadorwu et al. and Bouaziz et al. [13,14] with minor modifications, the dried seeds were ground into a fine powder using a high-speed multifunction grinder (HC-1500Y) (Faithful Instrument (Hebei) Co., Huanghua, China). The ground powder was then sieved using an automatic electric vibrating sieve equipped with a 20-mesh stainless steel screen (0.850 mm nominal sieve opening) to obtain a uniform particle size. The sieved powder was collected and stored in airtight, food-grade containers at room temperature until further experimentation.

For biphasic extraction, 0.04–0.06 g of the sieved seed powder was transferred into microcentrifuge tubes, and 0.50 mL of aqueous methanol (CH_3_OH:H_2_O, 66:34, *v*/*v*) and 0.50 mL of chloroform were added. The samples were vortexed for 5 min and then centrifuged in a 5415 R centrifuge (Eppendorf, Enfield, CT, USA) at 14.8 × 10^3^ rpm at room temperature for 15 min. After centrifugation, three phases were obtained: a top polar phase (aqueous methanol), a middle solid phase (insoluble material), and a bottom organic phase (chloroform). The top brownish phase contained polar compounds dissolved in the aqueous methanol solution, the bottom phase contained non-polar compounds dissolved in chloroform, and the solid phase contained compounds that were not soluble in either solvent. The organic and aqueous phases were carefully transferred into separate microcentrifuge tubes and concentrated in a Savant SpeedVac SPD1030 Integrated Vacuum Concentrator (Thermo Fisher Scientific, Winston-Salem, NC, USA) at room temperature and 8 torr for 4–6 h. The mass of each phase was recorded to determine the extraction yield. All extractions were performed on a single seed batch (biological sample), with four independent replicate extractions (n=4); the aqueous and organic phases accounted for 15.2 ± 1.9% and 13.7 ± 0.85% of the total mass, respectively.

### 2.2. Spectroscopic Measurements

#### 2.2.1. UV-Vis Analysis

UV-vis data were acquired on an Agilent BioTek Synergy LX multimode reader UV-vis instrument (Agilent Technologies, Santa Clara, CA, USA). The range of λ was set at 220–620 nm. The aqueous extract was dissolved in phosphate buffer at pH 7.2. Two sets of samples were prepared, one in the range of 1.36–4.42 mg/mL, and a more concentrated set in the range of 2.04–12.2 mg/mL to show the peak at 518 nm more clearly.

#### 2.2.2. HPLC-Analysis

Chromatographic experiments were performed using Agilent Technologies 1260 Infinity II system (Agilent Technologies, Santa Clara, CA, USA) equipped with a refractive index detector (RID). Chromatographic separation was carried out in isocratic mode at a flow rate of 1.4 mL/min using HPLC-grade acetonitrile/purified water (75:25, *v*/*v*) as the mobile phase. The column and detector were maintained at 30 °C, with a 10 µL injection volume and a total run time of 20 min. Sugar quantification for each sample was based on three replicate injections, and calibration standards were also injected in triplicate to assess analytical precision.

Standard stock solutions (10 mg/10 mL) of fructose, glucose, and sucrose were prepared in acetonitrile/water (50:50, *v*/*v*) and used to generate external calibration curves, which were linear over the tested range (R^2^ ≥ 0.99 for each sugar). Three independent *Malakabi* Ajwa seed samples (0.025–0.028 g in 1 mL acetonitrile) were analyzed, yielding fructose, glucose, and sucrose contents of 0.0142 ± 0.0010, 0.0149 ± 0.0058 and 0.0127 ± 0.0041 g/g seed, respectively (mean ± SD, *n* = 3), corresponding to approximately 1.42%, 1.49%, and 1.27% (*w*/*w*).

#### 2.2.3. Nuclear Magnetic Resonance (NMR) Spectroscopy

All NMR data were acquired on a Bruker AVANCE NEO (Bruker Instruments, Billerica, MA, USA) 400 MHz spectrometer equipped with a 5 mm X-nuclei optimized double-resonance broadband probe. NMR samples were prepared by dissolving the dried aqueous extract in phosphate buffer (pH 7.4) prepared with 90% H_2_O and 10% D_2_O. The dried organic extract was dissolved in deuterated chloroform (CDCl_3_) containing 1% tetramethylsilane (TMS), which served as the internal chemical shift reference (δ_H_ = 0.00 ppm for the methyl protons).

##### One-Dimensional NMR Experiments (TopSpin 4.0)

1H spectra were acquired using a 1D-presaturation NOESY pulse sequence, with data processed using a line broadening (LB) of 0.1 Hz. One-dimensional selective TOCSY spectra were recorded using a homonuclear Hartmann–Hahn transfer pulse sequence, with the MLEV17 sequence applied for mixing [15,16]. The mixing time was 0.08 s, and 256 scans were collected. Data were processed with a line broadening of 1.0 Hz. ^31^P NMR spectra were acquired in proton-decoupled mode with 16,000 scans and processed with a line broadening of 12 Hz.

##### Two-Dimensional NMR Experiments (TopSpin 4.0)

Correlation spectroscopy (COSY) spectra were acquired using the standard non-phase-sensitive sequence for 2D homonuclear shift correlation [17,18]. Data were collected with a 2K × 256 matrix and zero-filled to 2K × 1K points. Processing employed QSIN window functions with the following parameters: F2 dimension-LB 1.0 Hz; and F1 dimension-LB 0.3 Hz, Gaussian broadening (GB) 0.1 Hz. Total correlation spectroscopy (TOCSY) spectra were acquired in phase-sensitive mode using a homonuclear Hartmann–Hahn transfer pulse sequence with MLEV17 mixing [19,20]. Data were collected with a 2K × 256 matrix, zero-filled to 2K × 1K, and processed using the following: F2 dimension-QSIN, LB 1.0 Hz, sideband suppression (SSB) 2 and F1 dimension-QSIN, LB 0.3 Hz, GB 0.1 Hz, SSB 2.

#### 2.2.4. LC-MS-MS Analysis

The data for the untargeted LC-MS-MS analysis were acquired twice where the sample of the dried aqueous extract was resuspended in Optima LC-MS grade water at 0.1 mg/mL concentration. LC-MS-MS analyses were performed on a Water Acquity Ultra-performance liquid chromatography system (Waters Corporation, Milford, MA, USA) coupled to a Thermo Scientific Q Exactive Plus (Thermo Fisher Scientific, Winston-Salem, NC, USA) mass spectrometer. A 3 µL sample injection was eluted at 0.4 mL/min from a Waters Acquity HSS T3 (100 mm × 2.1 mm) column using a binary solvent system consisting of 0.1% formic acid in water (mobile phase A) and 0.1% formic acid in acetonitrile (mobile phase B). The gradient program is as follows: 0–1 min 100% A, 1–11 min 100% A—0% A, 11–13.1 min 0% A—100% A, and 13.1–15 min 100% A. The MS was operated using the following parameters: source, heated electrospray ionization (HESI); polarity, positive/negative ion switching mode; capillary voltage, 2500 V; capillary temperature, 262.5 °C; sheath gas 50 L/min; and heater temperature at 425 °C. Full-scan mass spectra were acquired over an m/z range of 75–1125 amu.

Mass accuracy was maintained within 5 ppm, and the Q Exactive Plus Orbitrap mass spectrometer (Thermo Fisher Scientific Inc., Waltham, MA, USA) Thermo Scientific Xcalibur software was operated at a resolving power of 70,000 FWHM at *m*/*z* 200. Metabolite annotation was performed using Compound Discoverer 2.0 software in conjunction with the mzCloud, mzVault and METLIN databases, applying a mass tolerance of ± 5 ppm during database searching. Metabolite identification confidence was assigned according to the Metabolomics Standards Initiative (MSI) guidelines.

#### 2.2.5. GC-MS Analysis

First, 70 μL of hexane was added to 7.7 mg of dry organic phase. GC-MS data were acquired using a Shimadzu GC-MS-QP 2010S (Shimadzu Corporation, Columbia, MD, USA). A 2 µL injection of each sample was eluted from a DB-1HT MS capillary column (30.0 m length, 0.25 mm i.d., 0.10 µm film thickness) using helium as the carrier gas. The mass spectrometer was operated using the following parameters: source, EI; ionization energy, 70 eV; source temperature, 260 °C; and scan range, 40–600 amu. Several resources including NIST (National Institute of Standards and Technology) and the Human Metabolome Database (HMDB) were used to identify the fatty acid esters and other compounds. Identification was determined using retention time and fragmentation patterns.

#### 2.2.6. Fourier Transform Infrared (FT-IR) Spectroscopy

The spectrum was recorded using a Shimadzu IRTracer-100 (EN120V) (Shimadzu Corporation, Columbia, MD, USA) FT-IR spectrometer in transmission mode, with a resolution of 4 cm^−1^ and a scanning range of 400–4000 cm^−1^. A small amount of dried organic extract was placed directly onto the instrument lens for measurement.

## 3. Results

### 3.1. Aqueous Phase

**Figure 1:**
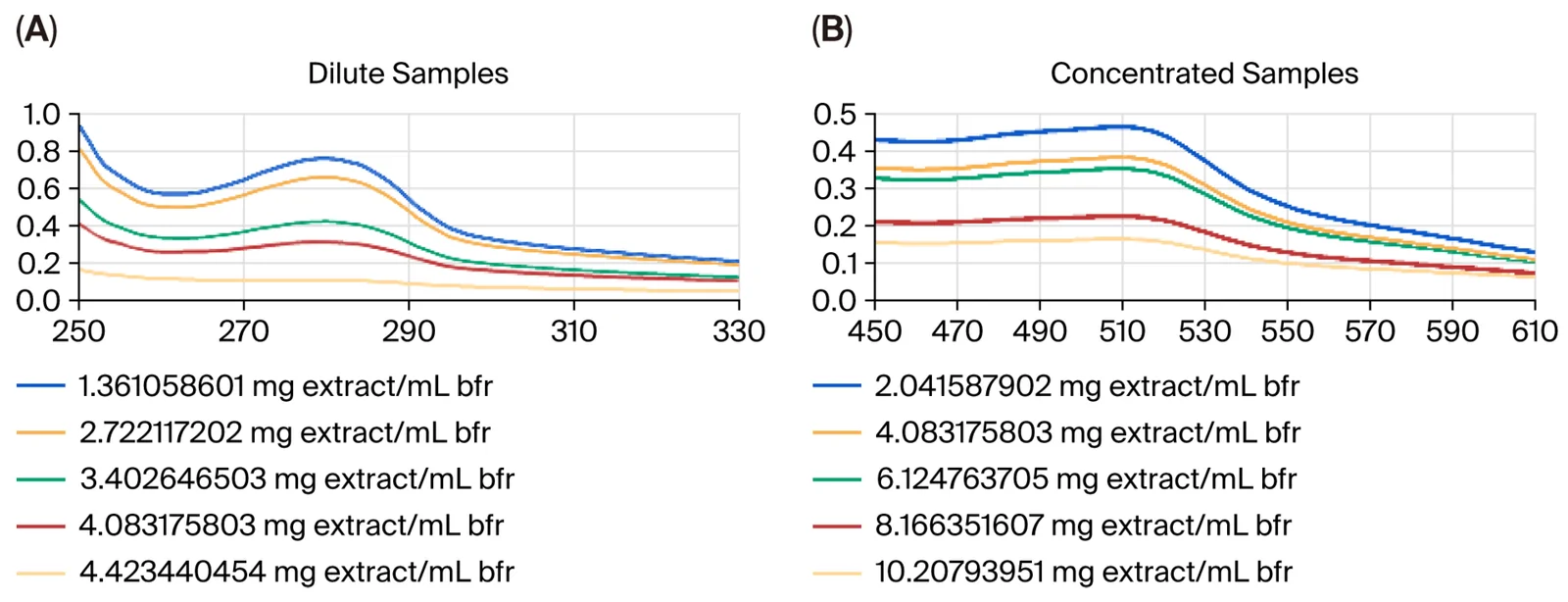
The UV-Vis spectra of the aqueous phase: (**A**) dilute samples where concentration range is 1.36–4.42 mg extract/1 mL buffer; (**B**) concentrated samples where concentration range is 2.04–10.21 mg extract/1 mL buffer.

Figure 1 shows the UV-Vis spectra of the aqueous extract at two concentration ranges: (A) 1.36–4.42 mg extract/mL buffer and (B) 2.04 –10.21 mg extract/mL buffer. The spectra display a distinct absorption band near 518 nm, which lies within the visible region commonly associated with anthocyanin-type pigments. Together with the LC-MS feature at *m*/*z* 432.097, these observations are consistent with the presence of a putative anthocyanin-type pigment, as further examined using tandem MS data, as described below.

HPLC-RID analysis of the aqueous phase showed that *Malakabi* Ajwa seeds contain roughly 1.4% fructose, 1.5% glucose, and 1.3% sucrose (*w*/*w*), with good reproducibility across three independent samples. The corresponding HPLC chromatogram of the aqueous phase and the related peak-area information are provided in the Supplementary Materials (Figure S2 and Table S1), where Table S1 reports the relative contributions of fructose, glucose and sucrose based on their peak areas.

**Figure 2:**
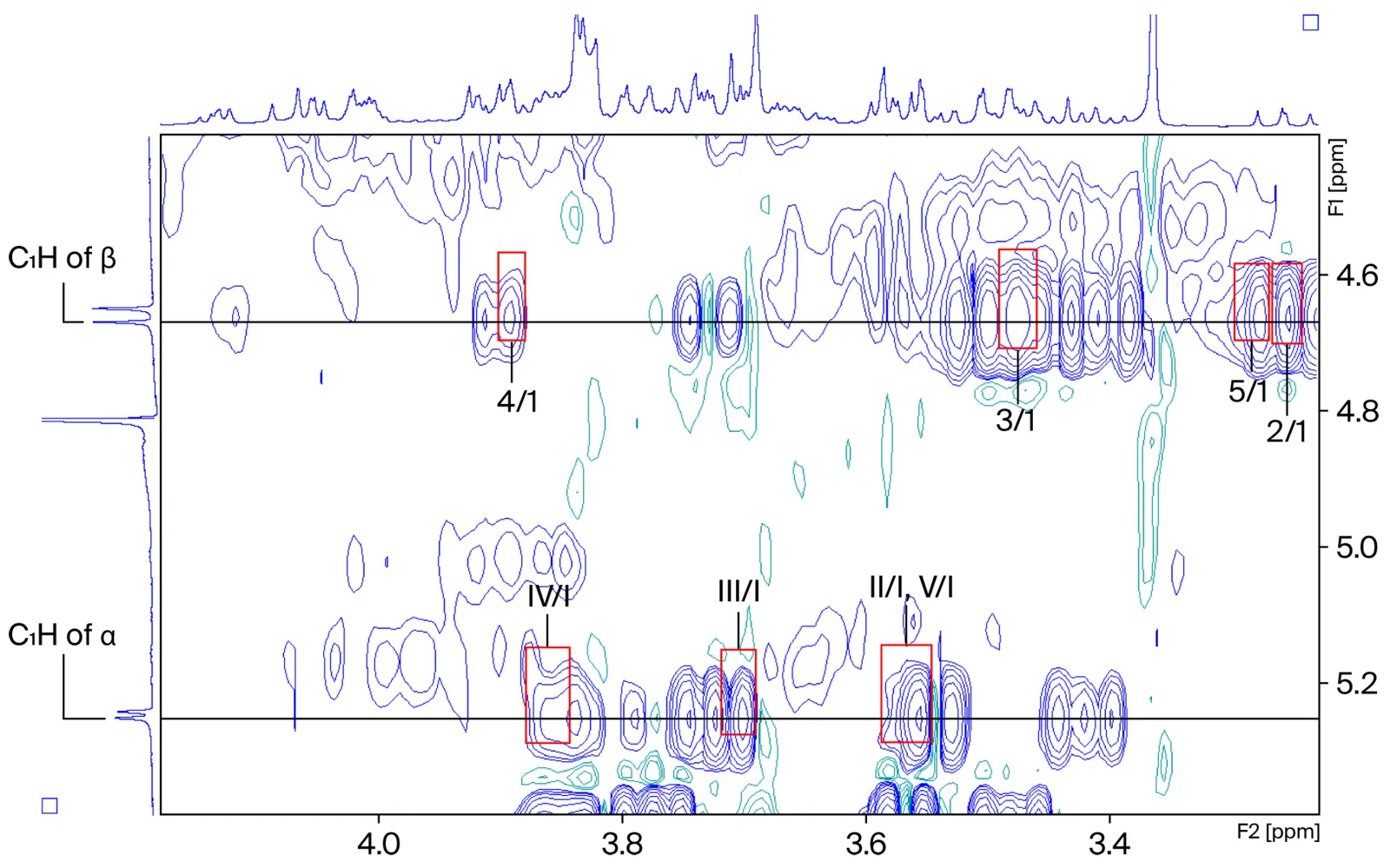
The section of the TOCSY spectrum shows the spin correlation between C_1_H proton and other protons in the α and β form of glucopyranose in the aqueous phase. The cross peaks in α form are labeled with Roman numbers and in β form with Arabic numbers.

2D ^1^H NMR provided structural data about sugars. Figure 2 presents a section of the TOCSY spectrum that shows the spin coupling correlation between C_1_H of the alpha and beta forms of glucopyranose and all protons except C_6_H. Figure S3 shows the full ^1^H NMR spectrum of the aqueous phase, where the relative intensity of the peaks corresponding to C_1_H of the alpha and beta forms of glucopyranose (C_1_H(β)/C_1_H(α)) is 1/0.82. The literature value for this relative abundance is 1/0.56 [21].

Figure S4 in the Supplementary Materials presents a section of the COSY spectrum that shows the spin couplings of the protons in the β form of glucopyranose, while Figure S5 presents another section of the COSY spectrum that shows the spin couplings between H1 and H6 protons in the α form. Table S2 in the Supplementary Materials reveals the chemical shift in the observed protons in the two glucopyranose forms along with the chemical shift in the literature [22,23].

**Figure 3:**
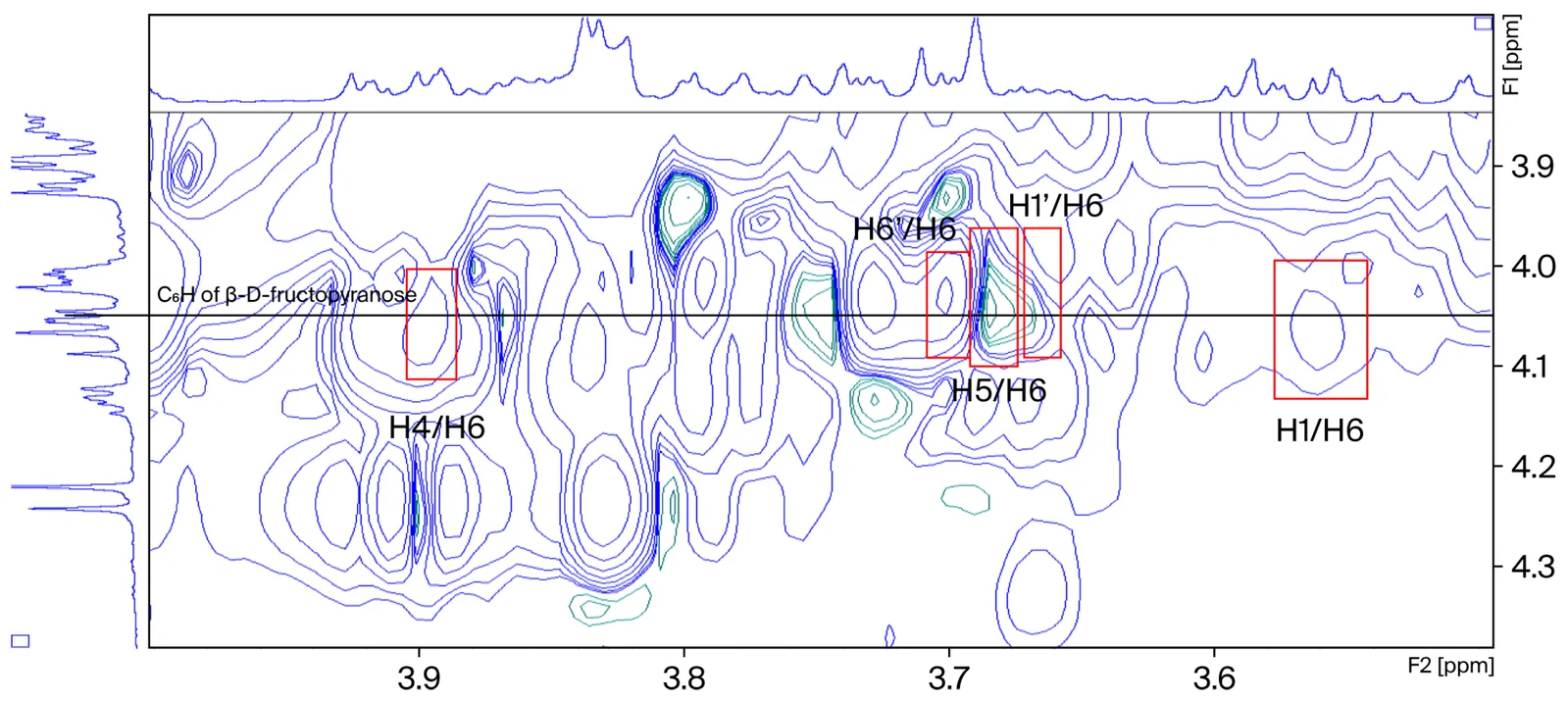
A section of the TOCSY spectrum that shows most of the spin correlations between C_6_H of ß-D-fructopyranose (at 4.04 PPM) and other protons.

**Figure 4:**
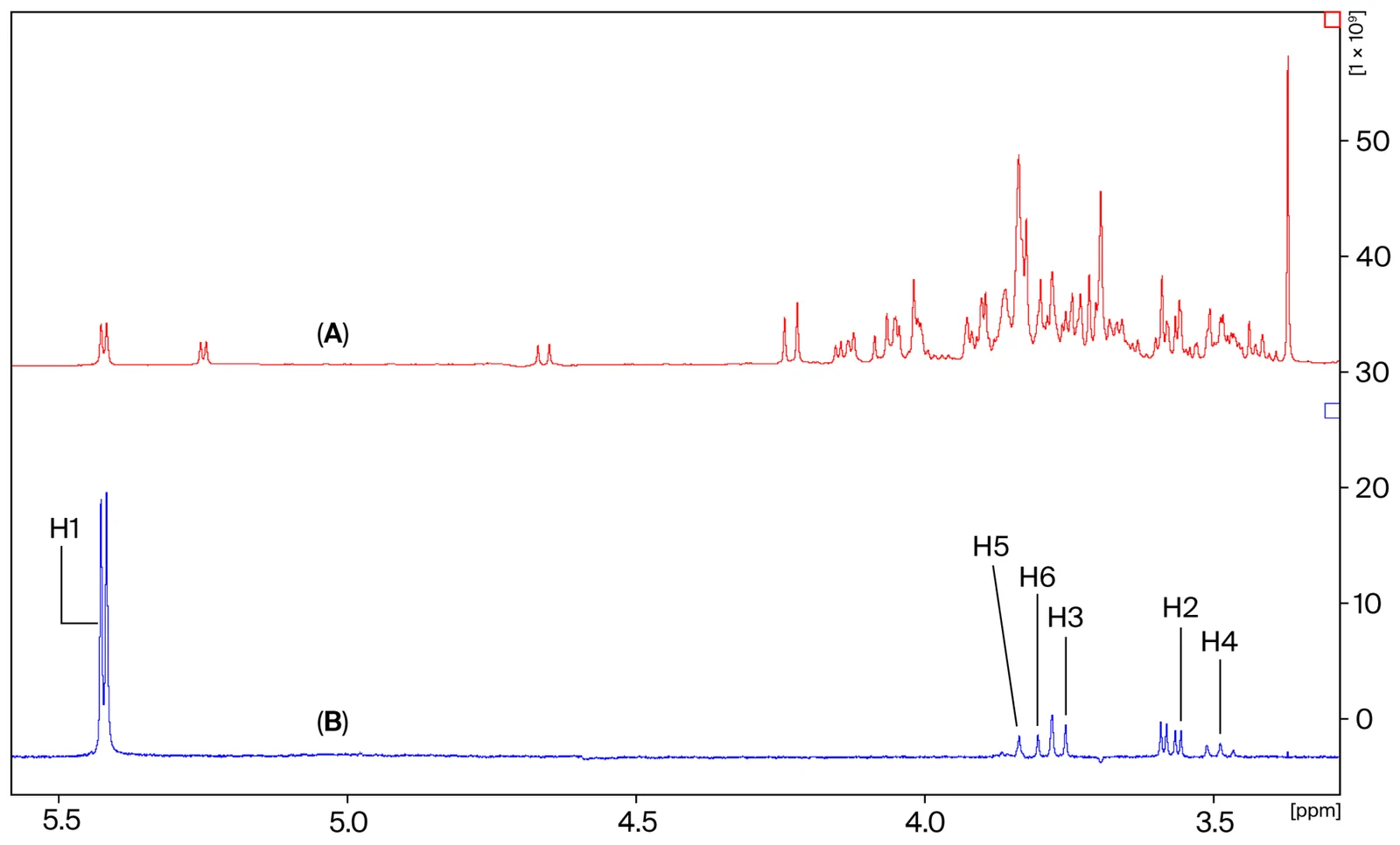
(**A**) Section of the ^1^H spectrum (400 MHz) of the aqueous phase. (**B**) is the 1D selective TOCSY spectrum when the peak at 5.45 PPM (corresponding to C_1_H of sucrose) was selectively excited. The spectrum shows the correlation between C_1_H and other protons in sucrose.

Figure 3 presents a section of the TCOSY spectrum that shows the spin-coupling correlation between C6H and other protons in the β-D-fructopyranose form of fructose. Table S3 in the Supplementary Materials shows the chemical shift in protons in this fructose form along with the chemical shift in the literature [23]. Figure 4 shows the 1D selective TOCSY spectrum that displays the spin coupling between C_1_H and other protons in sucrose, while Table S3 in the Supplementary Materials shows the chemical shift in protons in sucrose along with the chemical shift in the literature [24]. The section of the 2D TOCSY that displays the spin coupling in sucrose is shown in the Supplementary Materials (Figure S6).

**Figure 5:**
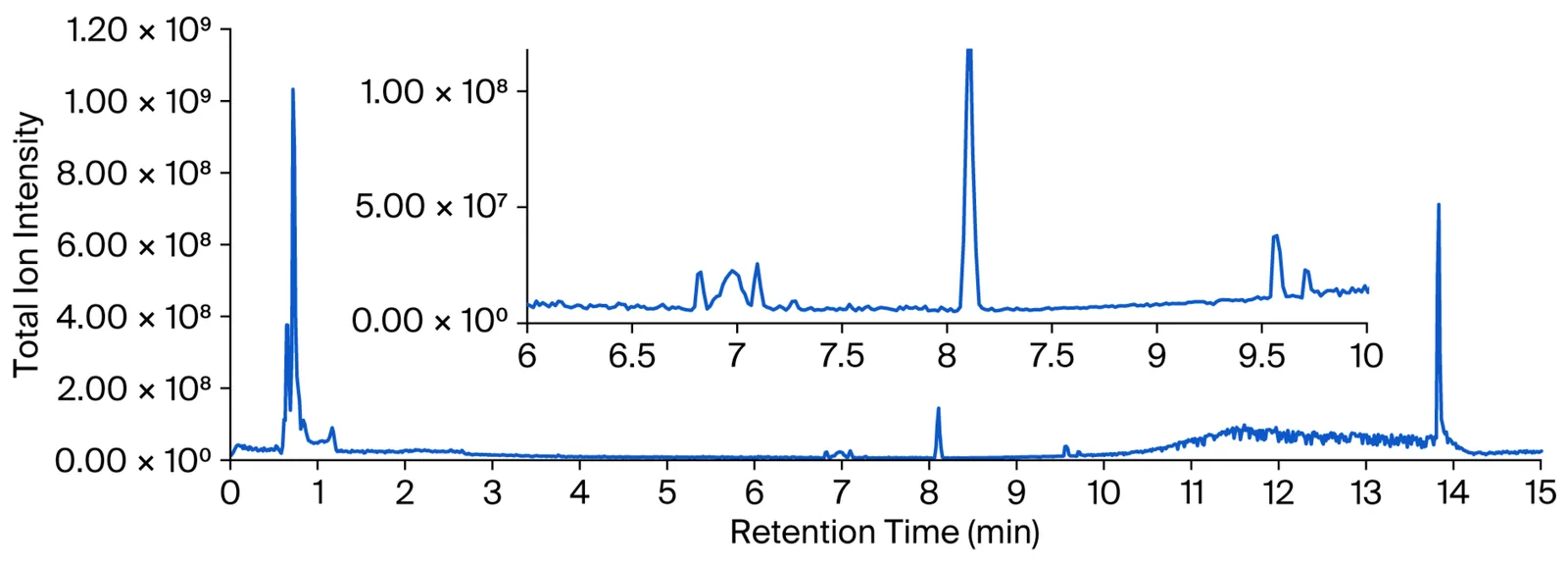
Mass chromatogram of the aqueous phase; inset shows the 6–10 min retention time region.

LC-MS data were analyzed for identification of several metabolites in the aqueous phase. Figure 5 shows a section of the mass chromatogram of the aqueous phase, where the peak at 8.11 min corresponds to a putative anthocyanin-type pigment. The full LC-MS chromatogram and the assignment of selected metabolites are shown in Figure S7A,B in the Supplementary Materials. Figure S8A–C displays the LC-MS chromatograms of the standards listed in Table 1.

The MS spectrum corresponding to the peak at 8.11 min (Figure S7A) shows an [M + H]^+^ ion consistent with an anthocyanin-type pigment previously reported for date seeds [25]; however, targeted MS/MS and comparison with authentic standards would be required for definitive structural confirmation. Table 1 lists the retention times of the [M + H]^+^ ions corresponding to leucine, phenylalanine, histidine, glucopyranose, fructose, and sucrose in the LC-MS data of the aqueous extract and, where available, the retention times of the corresponding standards. It also lists the retention time and [M + H]^+^ for the pigment peak and indicates whether ^1^H NMR signals of each compound were observed.

### 3.2. Organic Phase and ^31^P NMR Spectra

**Figure 6:**
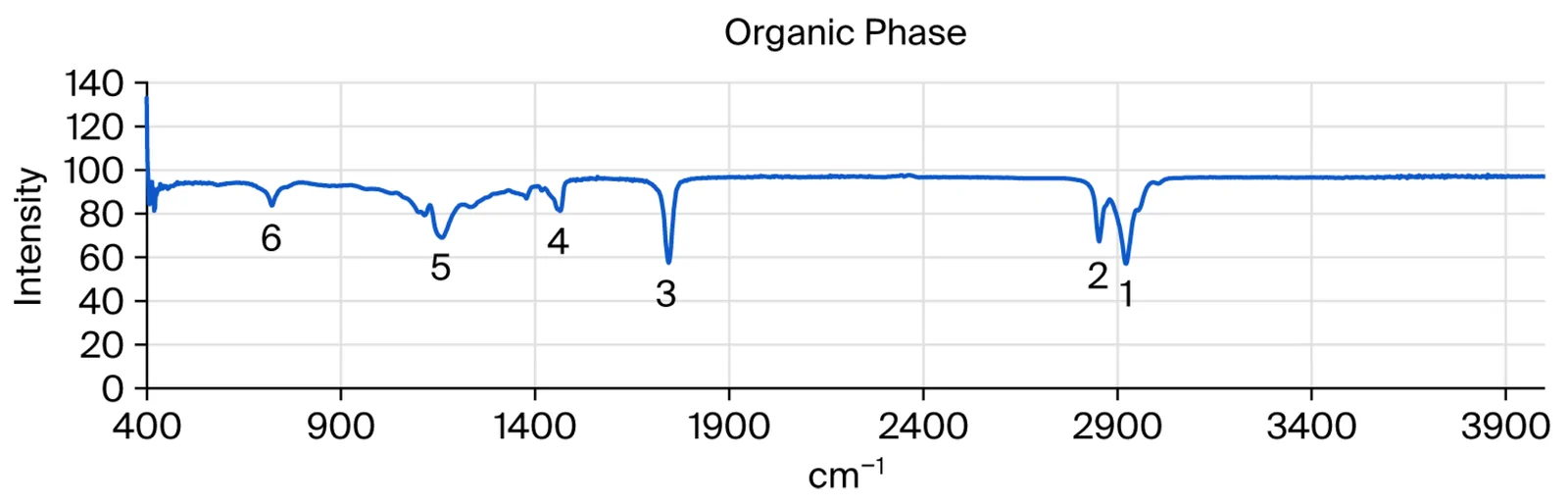
FT-IR peak assignments in the FT-IR spectrum of the organic phase. Peak #1 (C-H stretching-2945); #2 (C-H stretching-2850); #3 (C=O, 1743); #4 (C=C phenyl group, 1462); #5 (C-O, 1057); #6 (C-H bending, 721).

Figure 6 shows the FT-IR spectrum of the organic phase. The assignments of the peaks that are indicated in the figure legend are according to [26]. The spectrum lacks the peak corresponding to the O–H bond. This was used as initial evidence that the fatty acids in the organic phase are esterified and that the fatty acids in date seeds are naturally esterified. The GC/MS data that were acquired with and without esterification yielded identical MS spectra of the fatty acids, which presented further evidence for natural esterification. Thus, the presented GC-MS and NMR data of the organic phase were acquired without esterifying the fatty acids. In GC-MS data, the identification of the fatty acid esters Table 2 is achieved by comparing the experimental MS data with the MS spectra of fatty acid methyl ester in the NIST library match score, and this yielded a high percentage of confidence (86% or higher, Table 2). The confidence level of other compounds in Table 3 was obtained in the same way.

**Figure 7:**
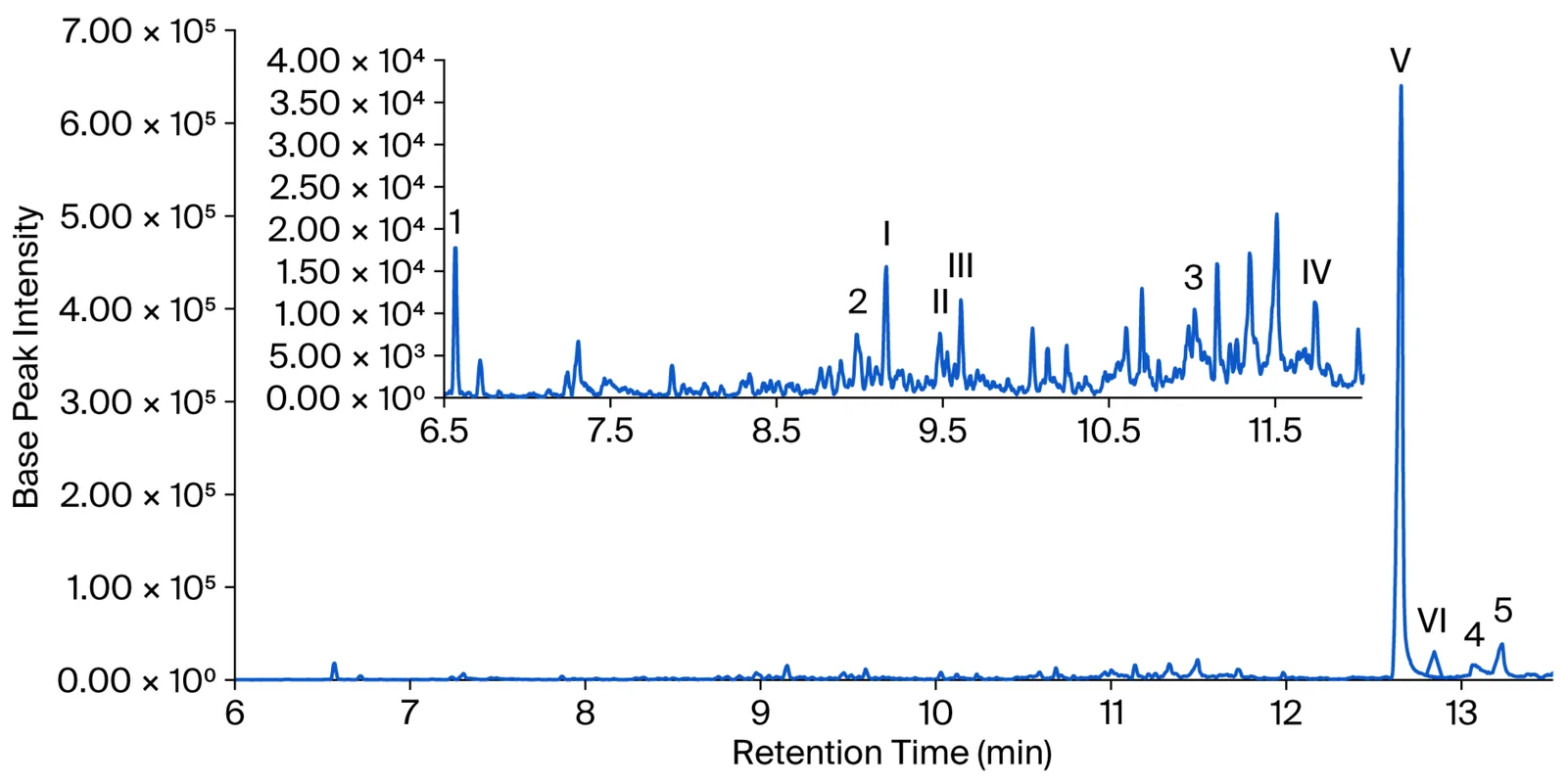
GC-MS chromatogram of the organic extract of date seeds. Peaks corresponding to esterified fatty acids are labeled with Arabic numerals, while other organic compounds are labeled with Roman numbers.

**Figure 8:**
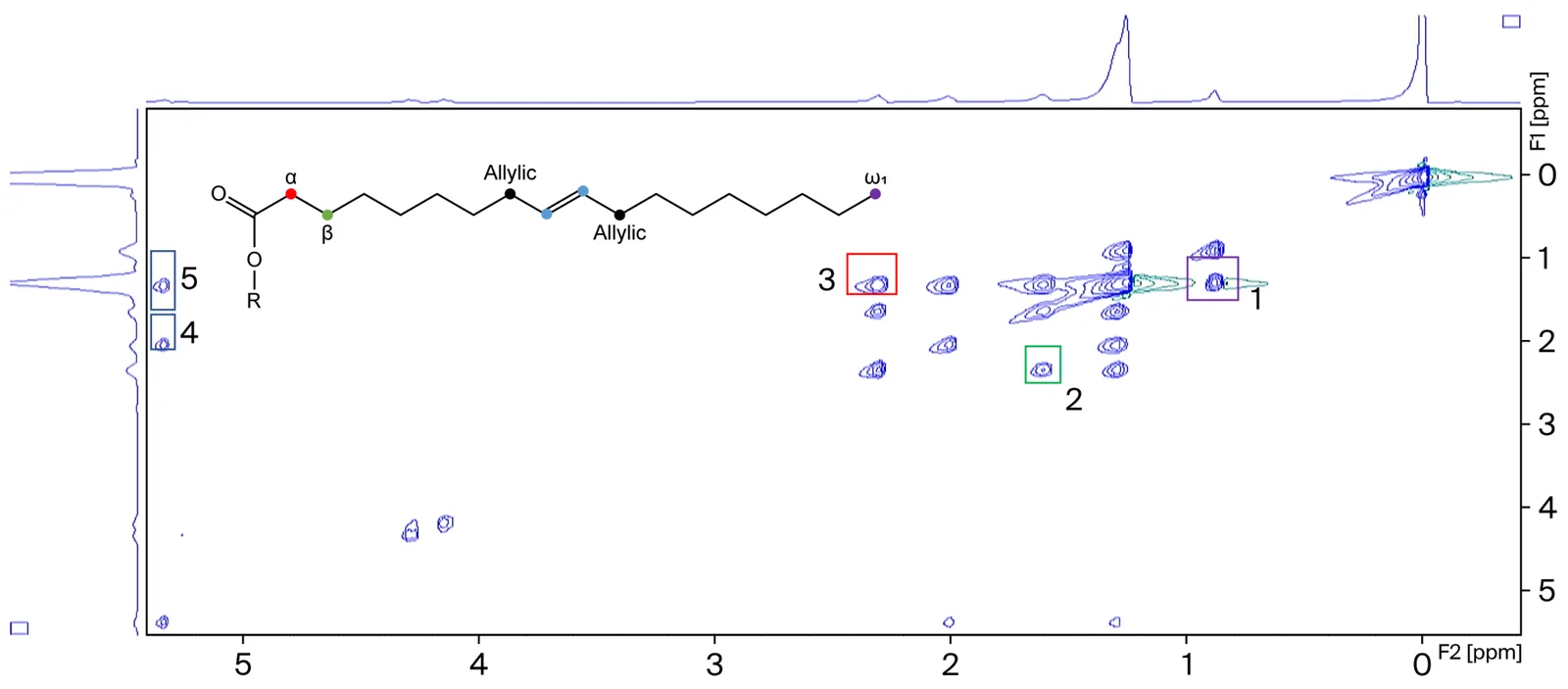
Section of the 2D TOCSY spectrum of the organic phase with identification of the cross peaks corresponding to the chain of the oleic acid whose structure is shown as an inset. The chemical shift in the cross peaks and their assignments are shown in Table 3.

**Figure 9:**
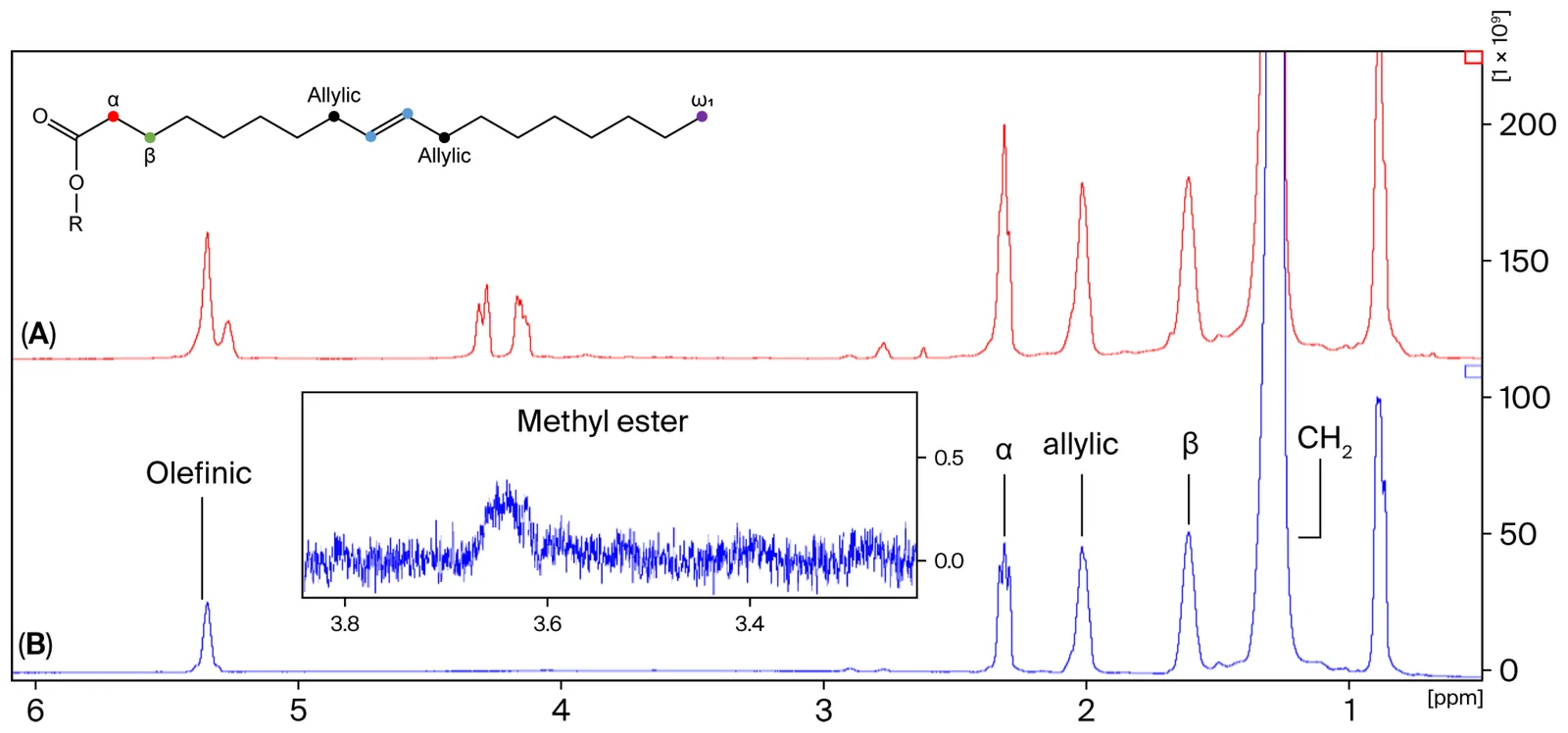
(**A**) ^1^H spectrum of the organic phase; (**B**) the 1D selective TOSCY spectrum corresponding to the excitation of the peak at 1.33 PPM which corresponds to all the CH_2_ groups in oleic acid except for α, β and the two allylic CH_2_ groups. The inset is a part of the spectrum in (**B**) where the intensity was increased to show the spin correlation to the methyl of the oleic acid methyl ester at 3.63 PPM.

GC-MS and NMR data provided identification of several metabolites in the organic phase. Figure 7 shows a section of the mass chromatogram of the organic phase (the full mass chromatogram is shown in Figure S9 in the Supplementary Materials). The peaks corresponding to the fatty acid esters are labeled with Arabic numbers, while other compounds in the organic phase are labeled with Roman numbers. Figur S10A, B displays the experimental and the database MS spectra that correspond to Lauric acid (RT 6.8 min) where its similarity score was 96% (Table 2). Table 2 presents the retention times and intensities of the GC-MS peaks, the tentative compound identifications based on fragmentation patterns, and the corresponding library match scores between the experimental and database mass spectra. In total, eleven compounds were identified using GC-MS data, six of which had a similarity score between 86 and 96%, and the others had 100% similarity scores (Table 2). The methyl esters of the following fatty acids were identified: lauric, myristic, palmitic, octacosanoic, and oleic acid. Figure 8 provides a section of the 2D TOCSY spectrum that shows the spin coupling correlation corresponding to the oleic acid chain (cross peaks 1 through 5); the structure of oleic acid is shown as an inset, and Table 3 lists the assignment of cross peaks 1 to 5. Figure 9A shows a section of the proton spectrum of the organic phase (the full ^1^H spectrum of the organic phase is shown in Figure S11 in the Supplementary Materials). The chemical shift in CH_2_ groups in oleic acid except for α, β, and the two allylic CH_2_ groups (see structure in Figure 9), is 1.29–1.33 PPM. Figure 9B is the 1D selective TOSCY spectrum corresponding to the selective excitation of the peak at 1.3 PPM, which shows all the expected spin coupling to all other groups in oleic acid including to the methyl group of the methyl ester at 3.63 PPM (inset of Figure 9). Figure S12 in the Supplementary Materials shows the proton-decoupled ^31^P spectra of the organic phase (A) and aqueous phase (B). The chemical shift is referenced to 85% with mass phosphoric acid when the chemical shift is set to 0 PPM. Based on the chemical shift in the ^31^P signals in both phases, it was determined that the main phosphorus compounds in both phases are orthophosphate monoester [27]. For ^31^P NMR experiments, the concentration of the aqueous and organic phases was very similar, and the number of scans in Figure S12A,B was the same. nonetheless, the S/N ratio in the aqueous spectrum was higher, indicating higher abundance of the orthophosphate monoester compounds in the aqueous phase.

**Table 3:** Chemical shift and assignments of the cross peaks corresponding to oleic acid chain in Figure 9. Olefinic protons are the protons in the C/C double bond.

Compounds	Chemical Shift in Cross Peak (ppm)	Cross Peak Number and Assignment
Oleic acid	1.23/0.9	(1) CH_2_/ω1
	2.34/1.61	(2) α/β
	1.33/2.34	(3) CH_2_/α
	2.05/5.33	(4) allylic/olefinic
	1.29/5.33	(5) CH_2_/Olefinic

## 4. Discussion

The metabolite profile of *Malakabi* Ajwa date seeds revealed a complex mixture of primary and secondary metabolites, including sugars, amino acids, fatty acid esters, and phosphorus-containing compounds. This complexity supports the use of biphasic extraction, in which metabolites were partitioned into aqueous and organic phases and each phase was analyzed separately using appropriate analytical platforms. Compared with a conventional monophasic 70–80% methanol in water extraction used for broad-coverage profiling of polar and semi-polar plant metabolites, this biphasic strategy is more efficient for capturing both polar and non-polar components in a single workflow [28,29]. Previous studies on date seeds have similarly reported carbohydrates, lipids, and phenolic compounds as major constituents of date seed biomass [30,31]. The present findings, however, extend these observations by providing structural confirmation of glucopyranose and fructopyranose using 2D NMR, and the identification of the phosphorous compounds in the *Malakabi* Ajwa date seeds using ^31^P NMR. One limitation of this study is that all four extraction replicates were performed on a single batch of *Malakabi* Ajwa date seeds. While these technical replicates showed that the extraction and analytical workflow is reproducible, they did not account for biological variability among independent seed samples. In future studies, we plan to include seeds from multiple date palm varieties and from plants grown under different environmental and soil conditions, so that variation in metabolite profiles across biological replicates can be more fully assessed.

### 4.1. Aqueous Phase Metabolite Profile

Glucose, fructose, and sucrose were identified as the major carbohydrates in the aqueous fraction. These sugars constitute important metabolic energy reserves and are consistent with previous reports describing date seeds as a potentially valuable ingredient in animal feed and functional food formulations because of their energy content and fermentable carbohydrate composition [5]. The relative integration of the anomeric ^1^H NMR signals (C_1_H) for glucopyranose gave a C_1_H, β/C_1_H, α ratio of 1/0.82, indicating a higher abundance of the β form under our conditions. This trend is conceptually consistent with the natural abundance of the two anomers reported in [21], where the corresponding ratio was 1/0.56, although the absolute values differ. The discrepancy may reflect differences in solution conditions, as the data were acquired for glucose in water and D_2_O, whereas our measurements were performed in phosphate buffer at pH 7.2 (90% H_2_O/10% D_2_O), and our experiments were not explicitly optimized for fully quantitative NMR integration.

### 4.2. Organic Phase

The FT-IR, GC-MS and NMR data together are consistent with fatty acids in *Malakabi* Ajwa date seeds being predominantly present in esterified forms rather than as free fatty acids. This suggests that the lipids occur mainly as storage molecules, which may contribute to oxidative stability and could enhance the potential industrial value of date seed extracts for food, cosmetic, and nutraceutical applications. The dominance of fatty acid methyl esters in the organic phase, including esters of oleic, palmitic, myristic and lauric acids, is a notable observation. Oleic acid is of particular interest as a major monounsaturated fatty acid that has been associated with cardiometabolic and anti-inflammatory benefits in other studies [32,33], although our work did not assess biological activity. The predominance of lipid metabolites in the organic phase is in line with previous reports describing date seed oil as rich in both unsaturated and saturated fatty acids [30,31].

Recent metabolomics studies have likewise highlighted the lipid richness of date seeds. Alsuhaymi et al. reported that seeds from several date cultivars, including Ajwa, are enriched in long-chain fatty acids and related hydrocinnamic acids compared with the corresponding fruit flesh, based on combined LC-MS and GC-MS profiling. Farag et al. [31] showed that roasting date pits used as coffee substitutes increases the relative abundance of fatty acids and generates characteristic volatile compounds, further underscoring the importance of lipidic constituents in date seed and pit matrices. Our GC-MS and NMR results for unroasted *Malakabi* Ajwa seeds complement these studies by providing detailed structural information on specific fatty acid esters in the organic phase and by demonstrating how a biphasic extraction can isolate these lipid metabolites for targeted characterization.

### 4.3. ^31^P NMR

Based on the chemical shifts in the ^31^P signals in the spectra of the aqueous and organic phases (Figure S9, Supplementary Materials), the main phosphorus compounds in both phases are orthophosphate monoesters. The predominance of orthophosphate monoesters revealed using ^31^P NMR is consistent with the central roles of phosphate-containing metabolites in energy storage and membrane-related metabolism in plant tissues. Our findings align with similar work carried out in our laboratory on beetroot extracts, where orthophosphate monoesters and diesters were identified as the major phosphorus species in both aqueous and organic phases, in agreement with published ^31^P NMR studies of plant matrices [13]. The higher relative signal intensity observed in the aqueous phase of date seeds suggests preferential partitioning of these phosphorus compounds into the polar phase, consistent with their ionic nature. Phosphorylated metabolites are important components of seed physiology because they participate in energy transfer, biosynthesis, and germination-related metabolic processes, particularly during the early activation of metabolism after imbibition. This is in line with the study by Weitbrecht et al., who suggested that the onset of seed germination is driven by the rapid activation of primary metabolic pathways (such as carbohydrate and energy metabolism) and their regulatory networks, which collectively support the transition from a quiescent dry seed to a metabolically active, germinating seed [34,35].

### 4.4. Complementary Analytical Approaches

Taken together, these results highlight the complementary roles of the analytical platforms used. GC-MS was found to be most effective for profiling volatile and semi-volatile constituents in the organic phase, including fatty acid esters and other low-polarity compounds that were not detected with LC-MS. HPLC provided quantitative information on major sugars in the aqueous phase, while LC-MS offered high sensitivity for additional polar metabolites such as amino acids and the putative anthocyanin-type pigment, which lay outside the practical detection range of GC-MS under our conditions. NMR spectroscopy contributed orthogonal structural information and enabled direct observation of abundant metabolites such as sugars and orthophosphate monoesters, although signal overlap and limited sensitivity restricted the identification of minor components. UV-Vis and FT-IR supplied rapid, global signatures of chromophores and functional groups, thereby supporting and contextualizing the chromatographic and mass-spectrometric findings.

## 5. Conclusions

A major outcome of this study is the demonstration that no single analytical platform provides comprehensive metabolite coverage. LC-MS offered high sensitivity for detecting amino acids and the anthocyanin-type pigment but provided limited structural information. In contrast, NMR enabled direct structural characterization of glucose anomers, fructose, and sucrose, yet lacked sufficient sensitivity to unambiguously assign signals from low-abundance amino acids and the pigment. HPLC supplied quantitative data for the major sugars, complementing the structural assignments from NMR. GC-MS proved particularly effective for the analysis of fatty acid esters, while FT-IR, through the absence of free O–H stretching bands, indicated that these fatty acids are naturally esterified, an interpretation supported by both GC-MS and NMR data. Taken together, these findings underscore the value of a multipronged analytical strategy that integrates chromatographic and spectroscopic platforms to achieve robust metabolite profiling of *Malakabi* Ajwa date seeds.

## Figures and Tables

**Table 1:** Retention times (RTs) of experimental and standard ${\left[M+H\right]}^{+}$ signals for leucine, phenylalanine, histidine and sucrose in the LC-MS data of the aqueous extract. The experimental RT of the ${\left[M+H\right]}^{+}$ signal corresponding to the pigment is also included.

	Formula	Experimental [MH]^+^	Experimental RT/Min.	Standard RT/Min.	[M + H]^+^ of Standard	Confirmed by NMR	Confidence Level *
**Amino Acids**							
Leucine	C_6_H_13_NO_2_	132.102	2.51	2.49	132.1018	No	2
Phenylalanine	C_9_H_11_NO_2_	166.086	3.03	3.02	166.086	No	2
Histidine	C_6_H_9_N_3_O_2_	155.0695	0.60	0.61	156.076	No	2
Valine	C_5_H_11_NO_2_	118.081	0.70	ND	ND	No	3
Isoleucine	C_6_H_13_NO_2_	132.09	13.82	ND	ND	No	3
**Sugars**							
Sucrose	C_12_H_22_O_11_	342.123	0.85	0.88	343.123	Yes	1
Glucopyranose	C_6_H_12_O_6_	180.156	0.66	ND	ND	Yes	2
Fructopyranose	C_6_H_12_O_6_	180.16	0.66	ND	ND	Yes	2
**Pigment type**							
Anthocyanin	C_78_H_89_O_44_	432.097	8.11	ND	ND	No	3

* Confidence levels were assigned on a three-point scale (1 = highest confidence): Level 1, mass spectrum of an authentic standard available and identity independently confirmed by NMR; Level 2, mass spectrum of an authentic standard available but identity not independently confirmed by NMR; Level 3, no mass spectrum of an authentic standard available and identity not independently confirmed by NMR. ND, not detected.

**Table 2:** MS identification of compounds in the organic extract of date seeds.

Peak	Retention Time	Intensity Level	Compound	Library Match Score (%) *
1	6.83	1.3 × 10^4^	Methyl ester of lauric acid	96%
2	8.97	2.0 × 10^4^	Methyl ester of myristic acid	94%
I	9.04	9.6 × 10^3^	Methyl tetradecanote	88%
II	9.09	9.6 × 10^3^	Octadecane	100%
III	9.60	2.0 × 10^4^	Lignocerane	100%
3	11.13	1.6 × 10^4^	Methyl ester of palmitic acid	93%
IV	11.72	2.3 × 10^4^	Docosane	100%
V	12.65	1.4 × 10^5^	Isopulegol	100%
VI	12.72	2.8 × 10^4^	Propyl acetate	100%
4	12.79	1.5 × 10^4^	Methyl ester of oleic acid	94%
5	13.12	1.6 × 10^4^	Methyl ester of octacosanoic acid	86%

* Library match scores generated with GC-MS software for NIST/HMDB spectrum.

## Data Availability

The original contributions presented in this study are included in the article/Supplementary Materials. Further inquiries can be directed to the corresponding author(s).

## Supplementary Materials

The following supporting information can be downloaded at https://www.mdpi.com/article/10.3390/metabo16080552/s1, Figure S1. α and ß structures of D-glucopyranose with natural abundance; Figure S2. HPLC chromatogram of the aqueous phase; Table S1. Relative percentages are based on the three sugar peak areas in the HPLC-RID chromatogram; Figure S3. Complete 400 MHz 1H spectrum of the aqueous phase; Figure S4. The section of the COSY spectrum that shows the spin coupling of H1–H5 in the β from of glucopyranose; Figure S5. The section of the COSY spectrum that shows the spin coupling of H1–H6 in the α from of glucopyranose; Table S2. Chemical shift (δ) of the 1H NMR signals of the α and β forms of glucopyranose; Figure S6. The section of TOCSY the spectrum of sucrose shows the spin coupling between H1 and other protons.; Table S3. The chemical shift in the H1-H6 of sucrose and of ß-D-fructopyranose; Figure S7. (A) LC-MS spectrum of the aqueous phase showing the assignment of anthocyanin at retention time 8.1 min; (B) Assignment of the several compounds in the LC-MS spectrum; Figure S8. (A) LC-MS spectrum of sucrose; (B) LC-MS spectrum of histidine; (C) LC-MS spectrum of phenylalanine; Figure S9. Full GC-MS chromatogram of the organic phase; Figure S10. Experimental (A) and reference library (B) MS spectra of methyl ester lauric acid (RT:6.83); Figure S11. The 400 MHz 1H spectrum of the organic phase; Figure S12. Proton-decoupled 31P spectra of the organic phase (A) and aqueous phase (B).
