# Supplementary material for: Advantages of a Multi-Platform Workflow Integrating Biphasic Extraction, Chromatography, and NMR for Characterization of Metabolites in Extracts of Malakabi Ajwa Date Seeds
Source: Metabolites. 2026 Aug 4;16(8):552. doi: 10.3390/metabo16080552 (PMC13515640; doi:10.3390/metabo16080552)
Supplement: Supplementary file 1 [file metabolites-16-00552-s001.zip › metabolites-4425337-supplementary.pdf]

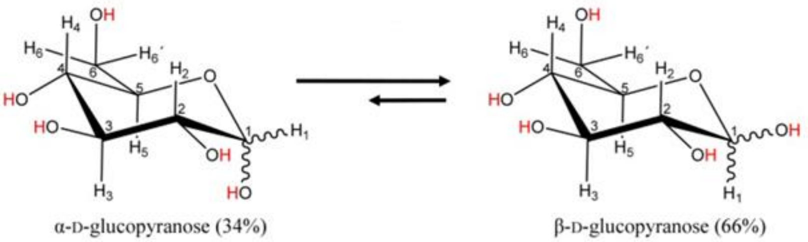

Figure S1. α and β structures of D-glucopyranose with natural abundance.

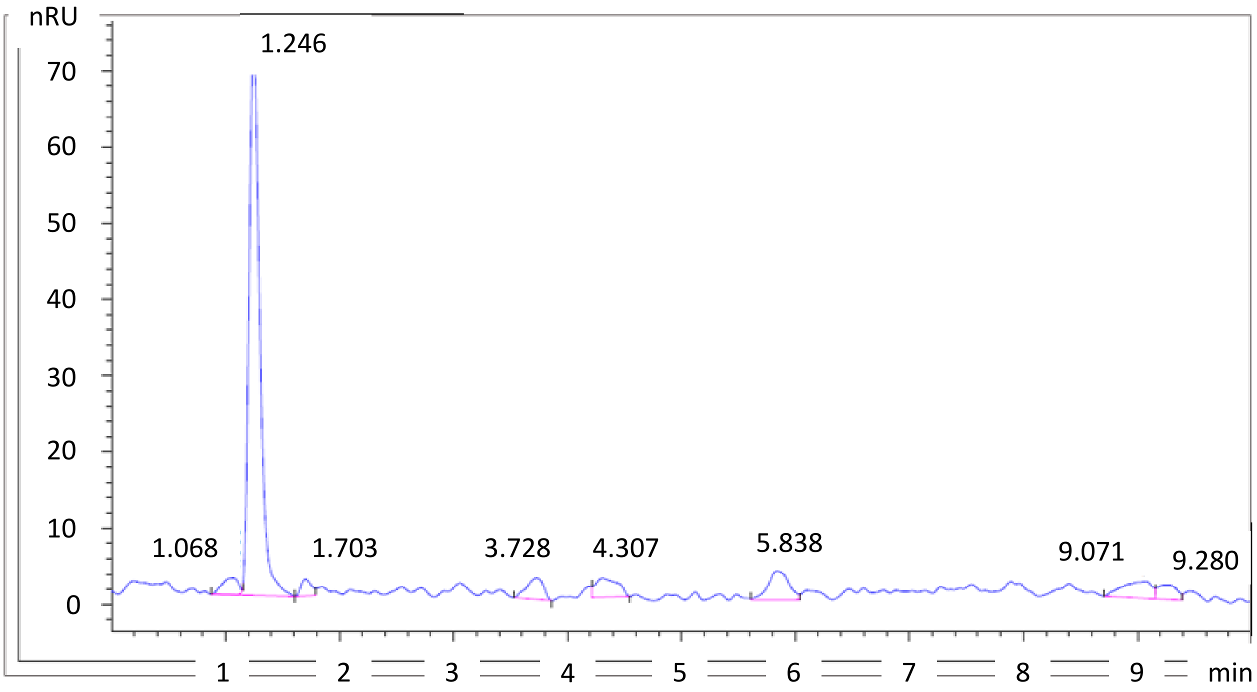

Figure S2. HPLC chromatogram of the aqueous phase.

Table S1. The retention times and percentages of sugars based on the HPLC chromatogram of the aqueous phase.

Type of sugar	Fructose	Glucopyranose	Sucrose
Peak's Area	531.278	744.528	636.045
Retention Time (min)	5.838	4.307	3.728
Percentage %	27.80	38.90	33.30

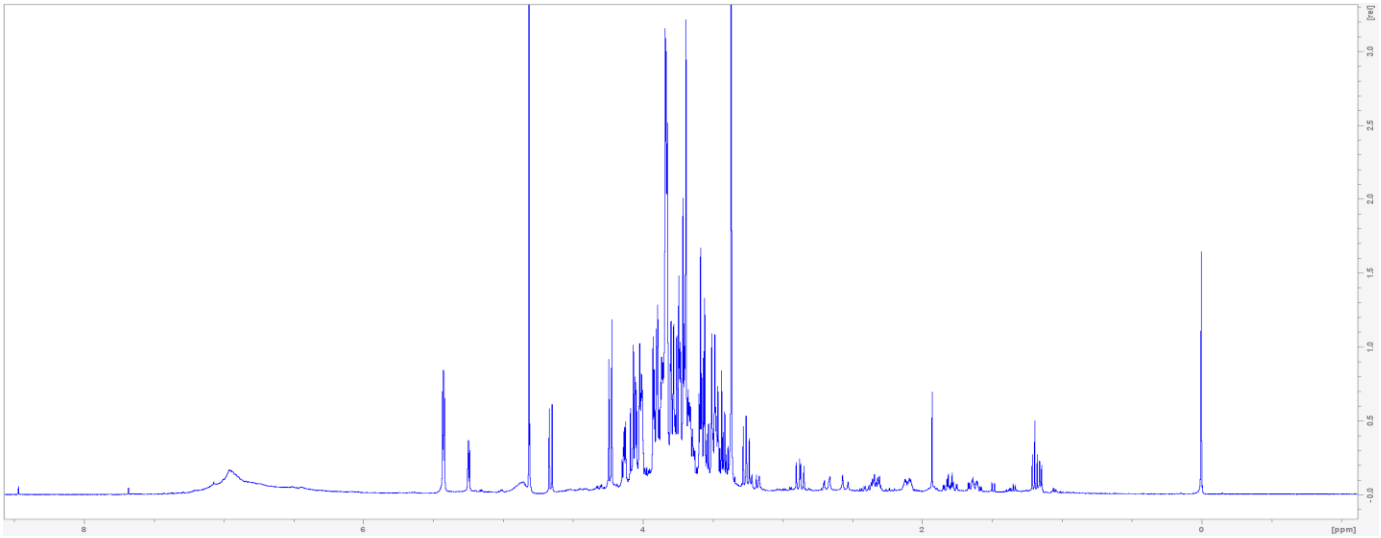

Figure S3. Complete 400 MHz ^{1}H spectrum of the aqueous phase

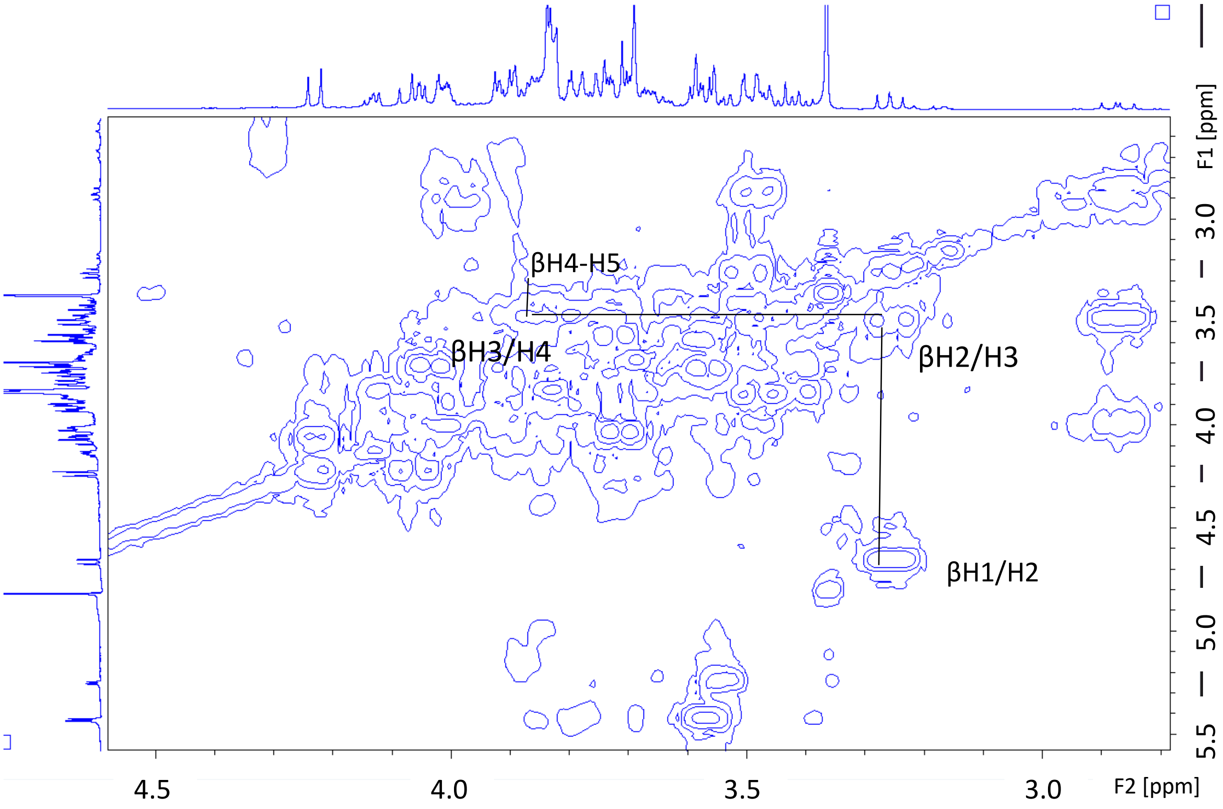

Figure S4. The section of the COSY spectrum that shows the spin coupling of H_1–H_5 in the β from of glucopyranose

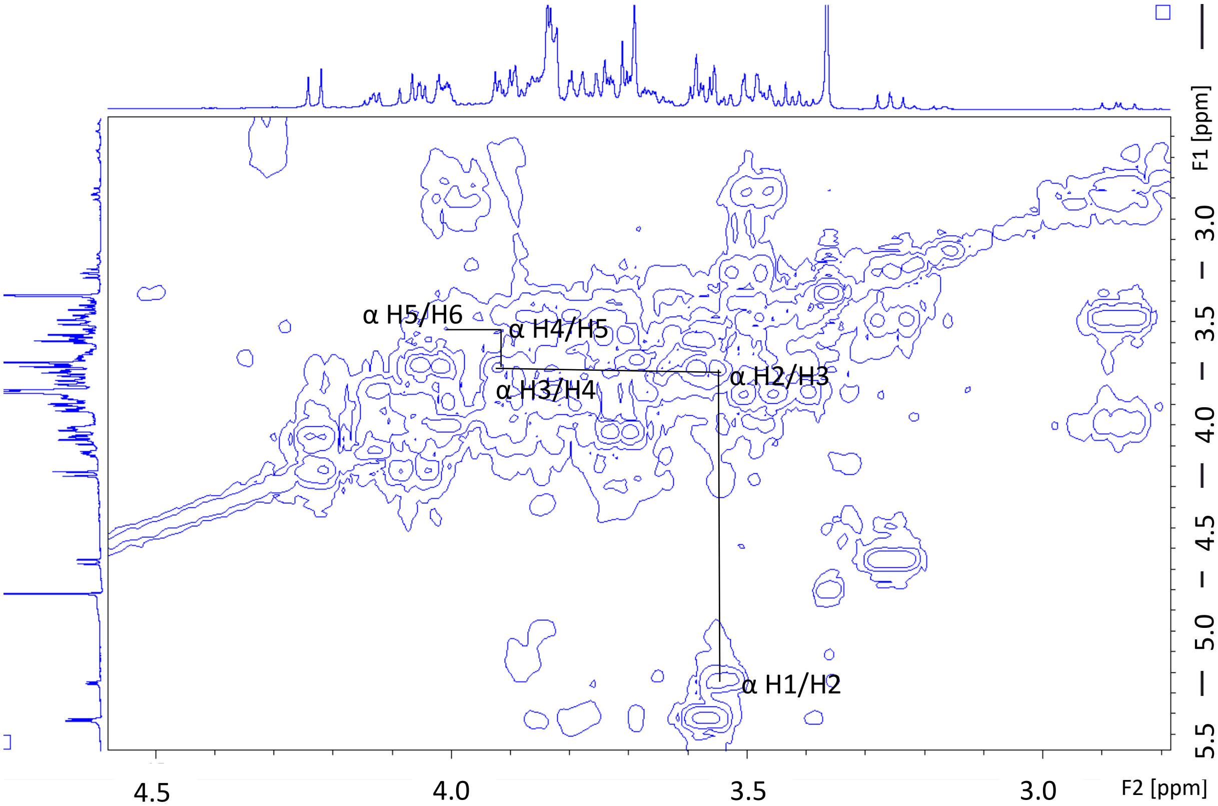

Figure S5. The section of the COSY spectrum that shows the spin coupling of H_1–H_6 in the *α* from of glucopyranose

Table S2. Chemical shift (δ) of the ^{1}H NMR signals of the *α* and β forms of glucopyranose.

Protons	δ of protons in β form of glucopyranose /δ	δ of protons in *α* form of glucopyranose /δ
H1	4.668/4.74	5.247/5.23
H2	3.259/3.37	3.554/3.63
H3	3.47/3.60	3.708/3.83
H4	3.89/3.92	3.86/3.92
H5	3.28/3.50	3.55/3.50
H6		4.01/3.91

(Pomin, V. H. (2012)

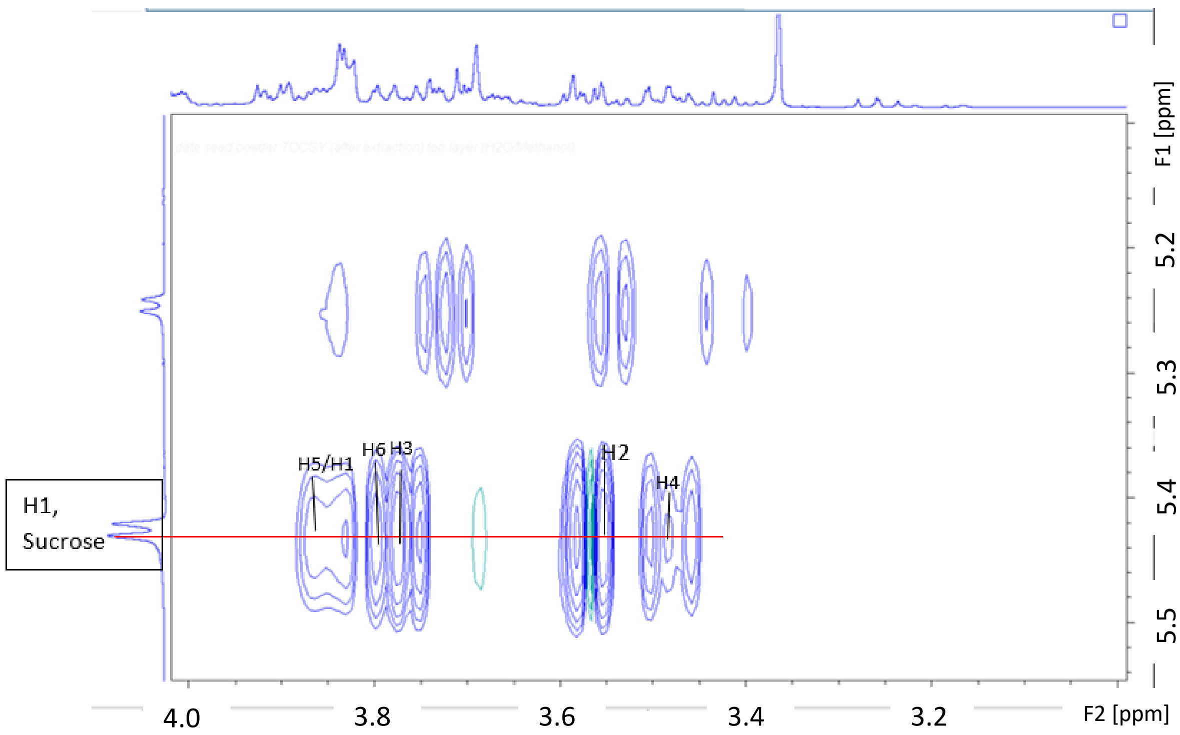

Figure S6. The section of TOCSY spectrum of Sucrose shows the spin coupling between H^1 and other protons.

Table S3. The chemical shift of the H_1-H_6 of sucrose and of β-D-fructopyranose.

Peaks of sucrose	δ(ppm)/literature δ (ppm)	β-D-fructopyranose	Experimental δ (ppm)/literature δ(ppm
H1	5.42/5.42	H1	3.73/3.71
H2	3.58/3.56	H1′	3.68/3.56
H3	3.75/3.76	H3	3.79/3.80
H4	3.50/3.48	H4	3.89/3.90
H5	3.86/3.86	H5	3.93/4.00
H6	3.83/3.82	H6	4.03/4.04
		H6′	3.73/3.71

(Barclay, T., Ginic-Markovic, M.,. (2012)

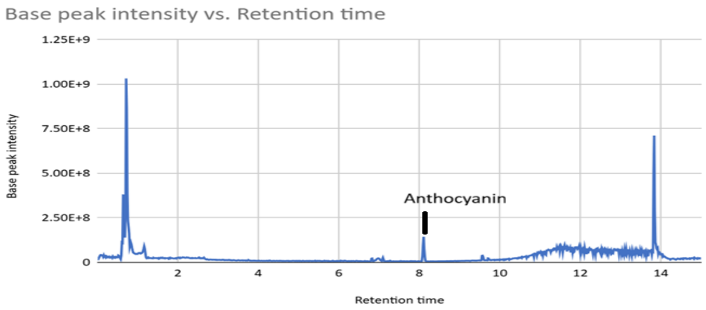

Figure S7A. LC-MS spectrum of the aqueous phase showing the assignment of anthocyanin at retention time 8.1 minutes

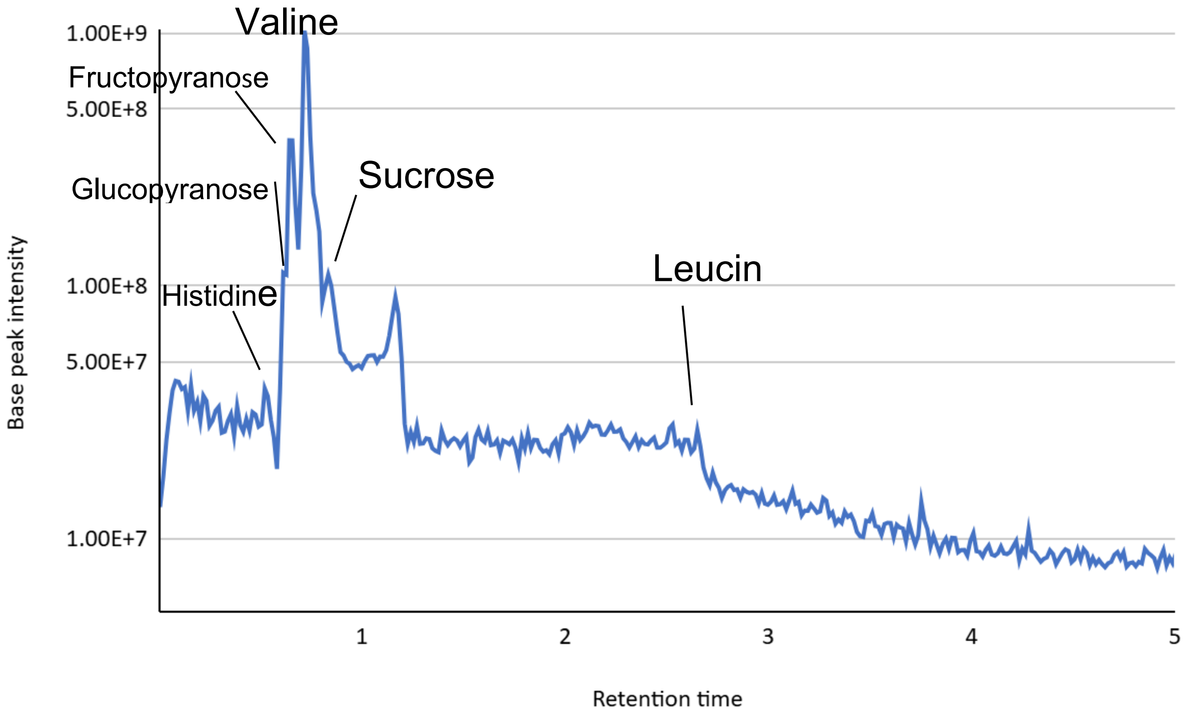

Figure S7 B. Assignment of the several compounds in the LC-MS spectrum

`

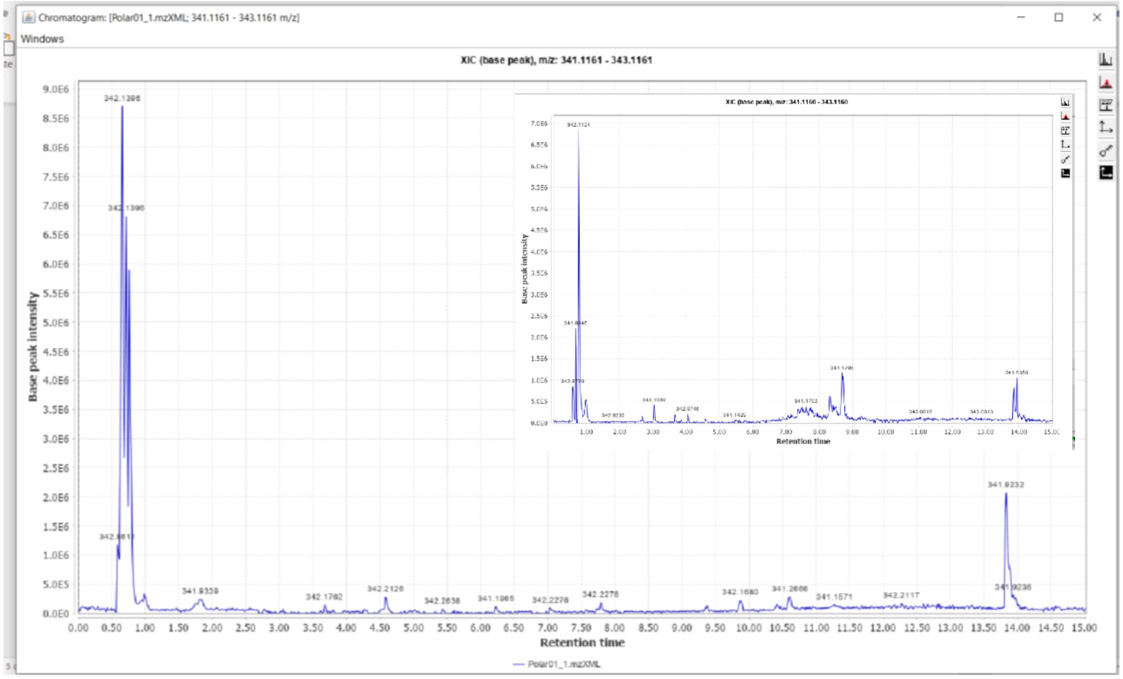

Figure S8A. LC-MS spectrum of sucrose.

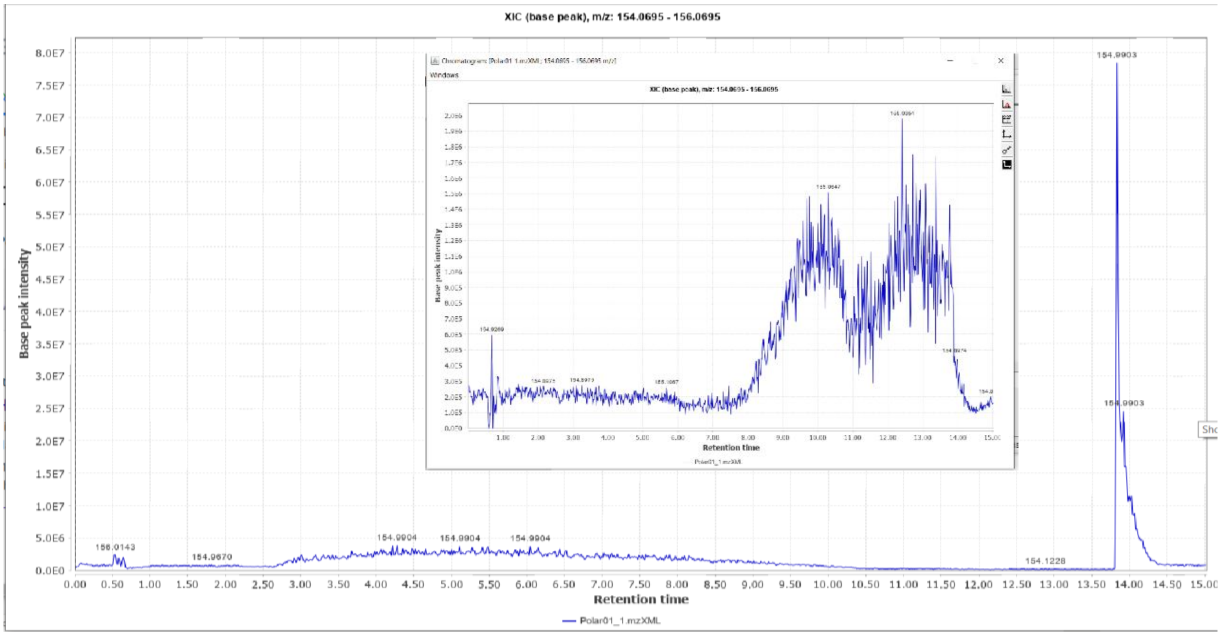

Figure S8B. LC-MS spectrum of histidine.

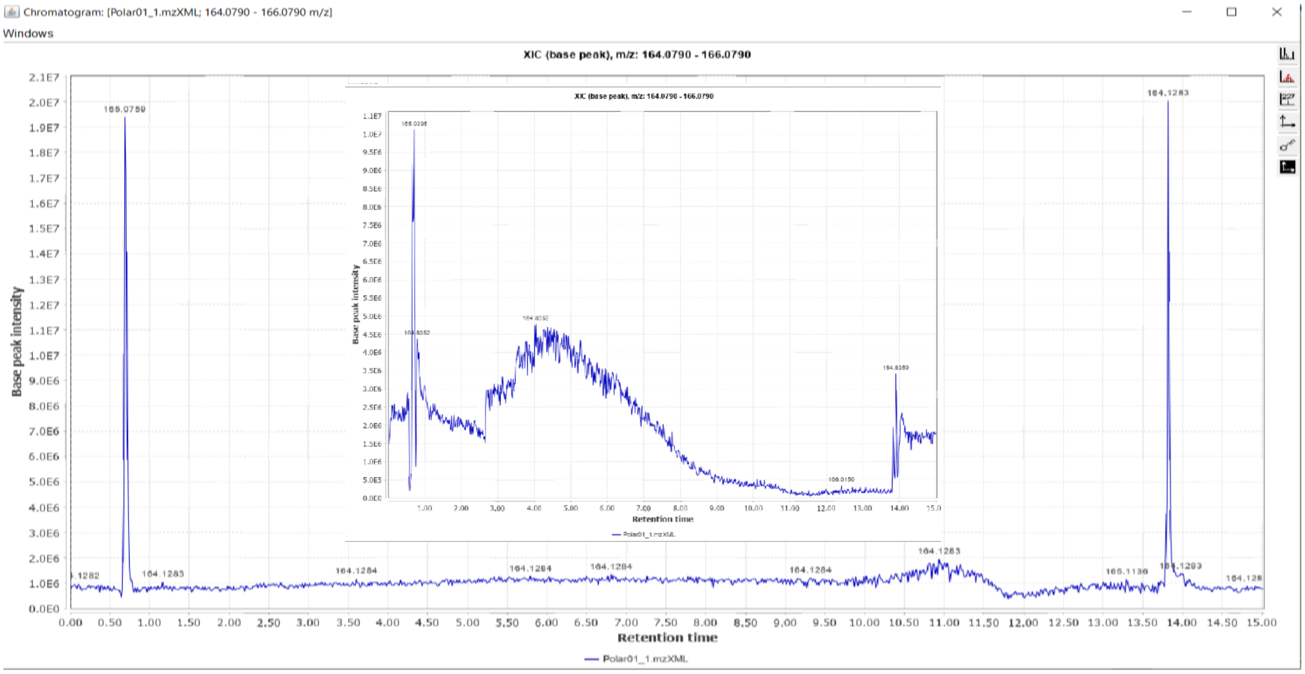

Figure S8C. LC-MS spectrum of phenylalanine

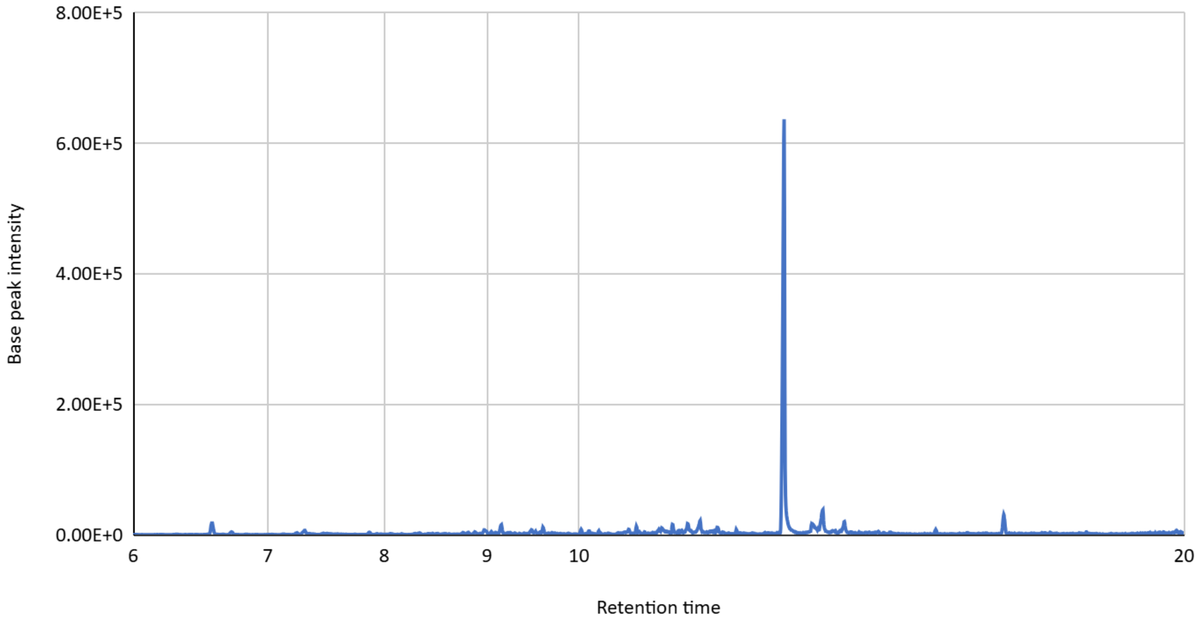

Figure S9. Full GC-MS chromatogram of the organic phase

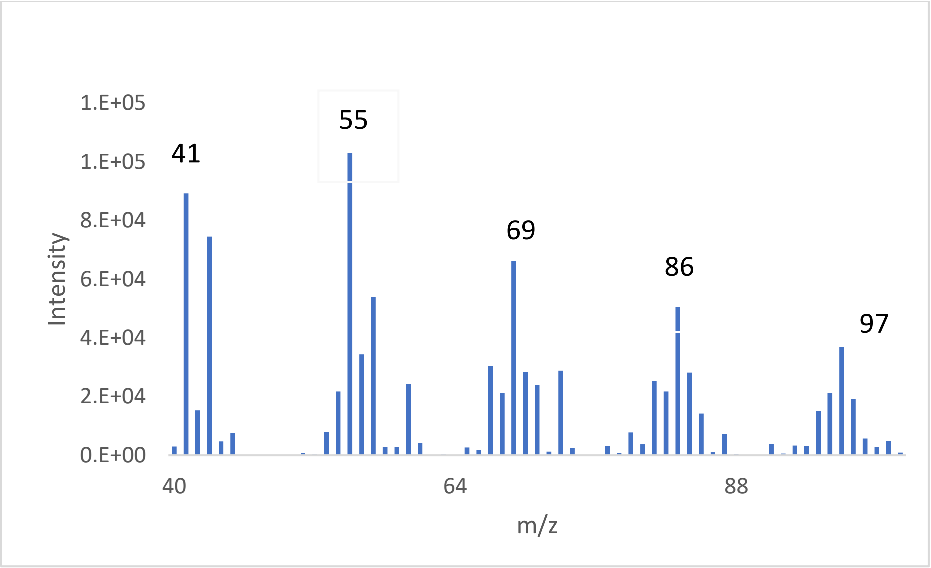

A

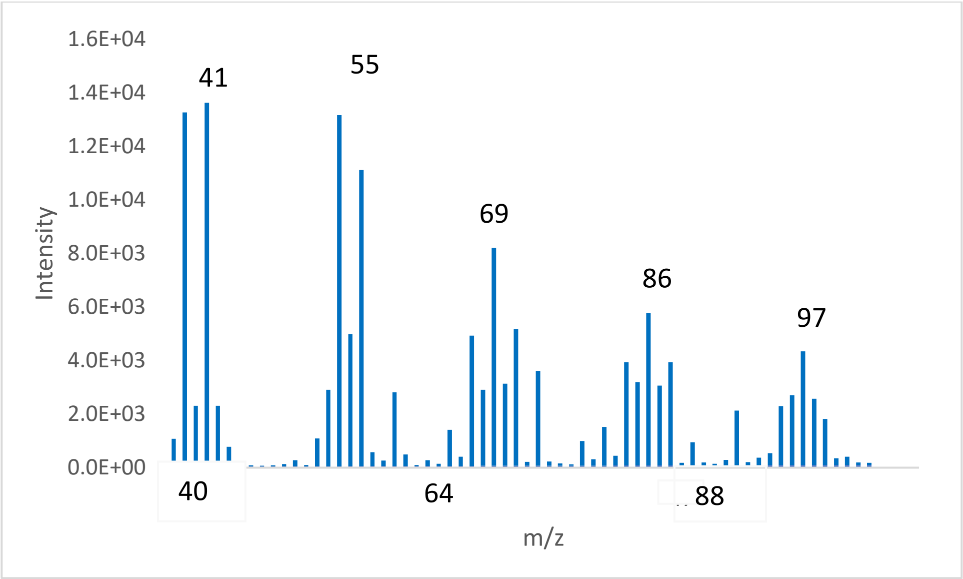

B

Figures S10. Experimental (A) and reference library (B) MS spectra of Methyl ester Lauric acid (RT:6.83)

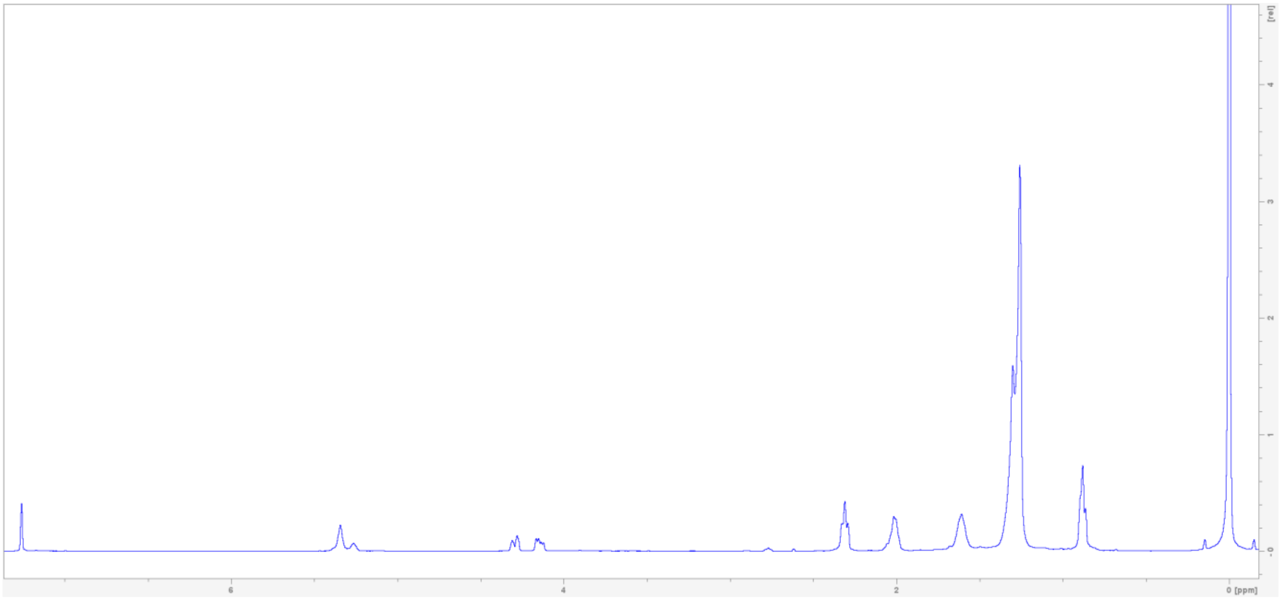

Figure S11. 400 MHz ^{1}H spectrum of the organic phase

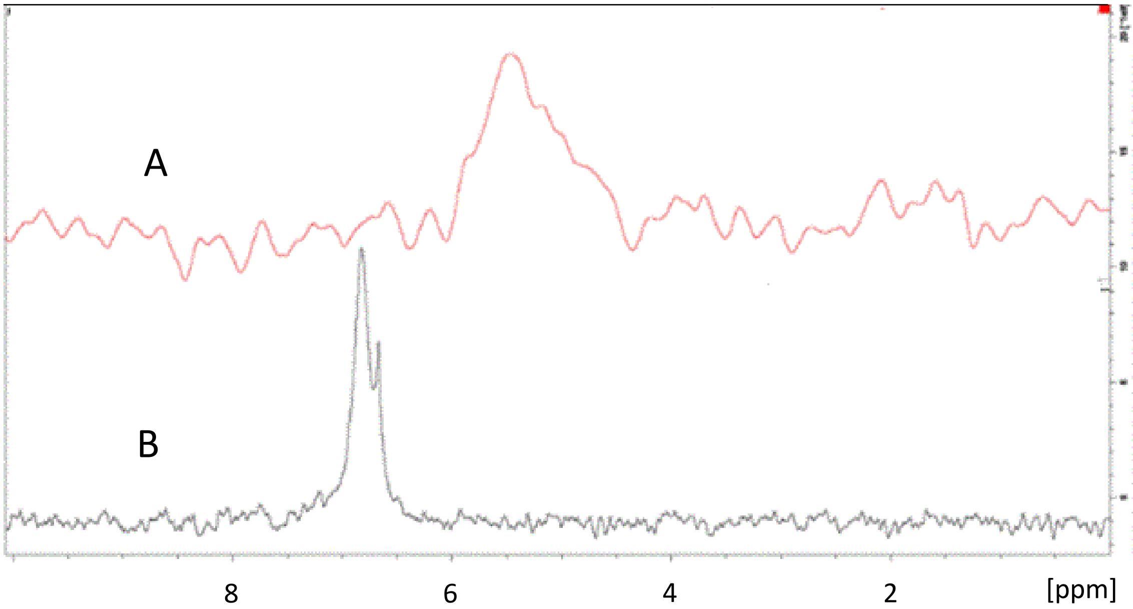

Figure S12. Proton-decoupled ^{31}P spectra of the organic phase (A) and aqueous phase (B)
